# Evidence against Zika virus infection of pets and peri-domestic animals in Latin America and Africa

**DOI:** 10.1099/jgv.0.001709

**Published:** 2022-01-25

**Authors:** Edmilson F. de Oliveira-Filho, Ianei O. Carneiro, Carlo Fischer, Arne Kühne, Ignacio Postigo-Hidalgo, Jorge R. L. Ribas, Peggy Schumann, Kathrin Nowak, Jan F. Gogarten, Xavier de Lamballerie, Filipe Dantas-Torres, Eduardo Martins Netto, Carlos Roberto Franke, Emmanuel Couacy-Hymann, Fabian H. Leendertz, Jan Felix Drexler

**Affiliations:** ^1^​ Institute of Virology, Charité-Universitätsmedizin Berlin, corporate member of Freie Universität Berlin and Humboldt-Universität zu Berlin, Berlin, Germany; ^2^​ Federal University of Bahia, Salvador, Brazil; ^3^​ Bahia State Agricultural Defense Agency, Salvador, Brazil; ^4^​ Labor Berlin, Charité Vivantes Services GmbH, Berlin, Germany; ^5^​ Epidemiology of Highly Pathogenic Microorganisms, Robert Koch Institute, Berlin, Germany; ^6^​ Viral Evolution, Robert Koch Institute, Berlin, Germany; ^7^​ Applied Zoology and Nature Conservation, University of Greifswald, Greifswald, Germany; ^8^​ Unité des Virus Émergents (Aix-Marseille University, IRD 190, Inserm 1207, IHU Méditerranée Infection), Marseille, France; ^9^​ Laboratory of Immunoparasitology, Department of Immunology, Aggeu Magalhães Institute, Oswaldo Cruz Foundation (Fiocruz), Recife, Brazil; ^10^​ Laboratoire National d'Appui au Développement Agricole/Laboratoire Central de Pathologie Animale, Bingerville, Côte d'Ivoire; ^11^​ Helmholtz Institute for One Health, Greifswald, Germany; ^12^​ Martsinovsky Institute of Medical Parasitology, Tropical and Vector-Borne Diseases, Sechenov University, Moscow, Russia; ^13^​ German Centre for Infection Research (DZIF), associated partner site Charité, Berlin, Germany

**Keywords:** Zika virus, flavivirus, serology, animal reservoir, zoonoses, antibody

## Abstract

Decades after its discovery in East Africa, Zika virus (ZIKV) emerged in Brazil in 2013 and infected millions of people during intense urban transmission. Whether vertebrates other than humans are involved in ZIKV transmission cycles remained unclear. Here, we investigate the role of different animals as ZIKV reservoirs by testing 1723 sera of pets, peri-domestic animals and African non-human primates (NHP) sampled during 2013–2018 in Brazil and 2006–2016 in Côte d'Ivoire. Exhaustive neutralization testing substantiated co-circulation of multiple flaviviruses and failed to confirm ZIKV infection in pets or peri-domestic animals in Côte d'Ivoire (*n*=259) and Brazil (*n*=1416). In contrast, ZIKV seroprevalence was 22.2% (2/9, 95% CI, 2.8–60.1) in West African chimpanzees (*Pan troglodytes verus*) and 11.1% (1/9, 95% CI, 0.3–48.3) in king colobus (*Colobus polycomos*). Our results indicate that while NHP may represent ZIKV reservoirs in Africa, pets or peri-domestic animals likely do not play a role in ZIKV transmission cycles.

The Zika virus (ZIKV) was first isolated from a sentinel rhesus monkey (*Macaca mulatta*) in Uganda during the 1950s and later reported in native non-human primate (NHP) species and in forest-dwelling *Aedes* mosquitos in Africa and therefore transmission in parts of Africa is thought to be maintained through interepidemic sylvatic cycles [1, 2]. ZIKV was not considered a major threat to human health [3, 4] until its emergence in Latin America, which was associated with severe congenital malformations including microcephaly [5]. According to evolutionary reconstructions, ZIKV was likely introduced into Brazil during 2013 [5] and infected approximately 8.5 million people during 2015 and 2016 [6]. Community protective immunity in urban centers likely contributed to cease of the epidemic [7].

Sylvatic transmission cycles may permit ZIKV maintenance until the pool of susceptible humans is replenished by birth and migration, potentially contributing to a resurge of intense urban ZIKV transmission cycles. Whether animal reservoirs play a role in ZIKV transmission in the Americas remains unknown. The available data on ZIKV infection of NHP in the Americas are inconclusive. Some South American NHP species, such as marmosets (*Callithrix jacchus*) and tamarins (*Saguinus labiatus*) were susceptible to ZIKV *in vivo* [8]. Low seroprevalence and low levels of neutralizing antibodies have been reported in capuchin monkeys in Northeastern and Central Brazil [9, 10]. Finally, ZIKV has been detected in NHP carcasses in the Southeastern region, but since contamination of the remains could not be excluded, the relevance of those findings still needs to be confirmed [11].

Several flaviviruses can infect pets and peri-domestic animals. For instance, experimental infection and serological evidence indicate circulation of West Nile virus (WNV) and Japanese Encephalitis virus (JEV) in cats and dogs [12–15] and of Wesselsbron virus (WSLV) in cattle, goat and sheep [15–21]. Virus detection by molecular methods, virus isolation and serological evidence substantiate that horses can be infected with WNV, Saint Louis Encephalitis virus (SLEV), JEV and Usutu virus (USV) [22, 23]. Livestock species, such as sheep and goats, were susceptible to ZIKV *in vivo* [24, 25], and although ZIKV-specific neutralizing antibodies were reported in sero-epidemiological studies in cattle and sheep from Brazil and in horses from French Pacific Islands [26, 27], only few flaviviruses beyond ZIKV were used in those studies to rule out potentially unspecific test results elicited by cross-reactive flavivirus antibodies. Exposure of vertebrates to ZIKV depends on the feeding preferences of the mosquito vectors. In Latin America, ZIKV vectors include predominantly *Aedes aegypti* and, to a lesser extent, *Ae. albopictus* [2, 28, 29]. In Africa, the invertebrate host range of ZIKV is not entirely known. However, major vectors likely include *Aedes aegypti formosus*, *Ae. africanus*, *Ae. albopictus*, *Ae. apicoargenteus*, *Ae. furcifer* and *Ae. vitattus* [28]. Whereas *Ae. aegypti* and, to a lesser extent, *Ae. albopictus* are highly anthropophilic, they are also known to opportunistically feed on other vertebrates, including rodents, cattle, sheep and dogs [29–34]. Those animals are reared in high numbers and in close proximity to humans and may thus be important components of (peri-)urban ZIKV transmission cycles. Therefore, it is currently unclear whether pets and peri-domestic animals play a role in the maintenance of ZIKV in Africa or the Americas.

Here we investigated ZIKV spread in pets (i.e. dogs and cats) and peri-domestic animals (i.e. equids, cattle, sheep and goats) sampled in northeastern Brazil, the 2015–2016 outbreak’s epicentre, and in wild NHP, pets and peri-domestic animals sampled in Côte d’Ivoire, where ZIKV has been documented in forest-dwelling mosquitos [4, 35].

In Brazil, equid, cattle, sheep, goat, dog and cat samples were collected in the northeastern state of Bahia during 2013–2018 within state routine veterinary surveillance activities or from dogs and cats presenting clinical signs, and in 2015 from dogs in the neighbouring state of Pernambuco for a *Leishmania* spp. serosurvey [36]. In Côte d'Ivoire, peri-domestic animals were sampled in 2012 and 2014. Unhabituated wild NHP such as king colobus (*Colobus polycomos*) and western red colobus (*Piliocolobus badius*) were sampled in the Taï National Park during 2006 and 2016 [37, 38]. Samples were also collected from a habituated group of sooty mangabeys (*Cercocebus atys*) that has been under observation since 2012 [37, 38]. Additionally, nine blood samples from deceased West African chimpanzees (*Pan troglodytes verus*) were included; this population of chimpanzees has been followed daily since 1979 and necropsy samples have been systematically performed on all dead individuals recovered, since the inception of the veterinary programme in 2002 [37, 39].

A total of 1416 sera of pets and peri-domestic animals from Brazil and 298 sera of dogs, peri-domestic animals and NHP from Côte d'Ivoire were screened for ZIKV antibodies using the NS1-based Electrochemiluminescence immunoassay (ECLIA) Elecsys Zika IgG (Roche, Penzberg, Germany). This double-antigen test enables multispecies testing because it does not require species-specific secondary antibodies, but it was only validated for human sera [40]. ECLIA-positive sera were further tested by a plaque reduction neutralization test (PRNT_90_) to identify anti-ZIKV neutralizing antibodies (Asian ZIKV lineage, strain H/PF/2013; used for samples from Brazil because of limited volume of available sera and African ZIKV lineage, strain MR766; used for samples from Côte d’Ivoire because of limited volume of available sera). Because cross-reacting antibodies against co-circulating flaviviruses can yield false-positive ZIKV test results [41, 42], we compared ZIKV PRNT_90_ endpoint titres with endpoint titres against co-circulating flaviviruses representing diverse serocomplexes, including Rocio virus (ROCV; strain UVE/ROCV/1975/BR/5P H34 675), Saint Louis Encephalitis virus (SLEV; strain MSI-7), Bussuquara virus (BSQV; strain BeAn 4073), Wesselsbron virus (WSLV; strain UVE/WESSV/UNK/ZA/SAH-177), Spondweni virus (SPOV; strain UVE/SPOV/UNK/ZA/SM-6 V-1s), Dengue virus 2 (DENV-2; strain Thailand/16681/84), Yellow Fever virus (YFV; strain 17D), and West Nile virus (WNV; strain NY-99). We used antigenic cartography to discern neutralizing antibody reactivity patterns [43]. Due to the lack of sufficient volume of serum samples, chimpanzee sera were only tested by PRNT_90_ and not by ECLIA.

PRNT_90_ was conducted in 12-well plates seeded 1 day before the infection with 1.6×10^5^ Vero FM cells for ZIKV, SPOV, YFV, WSLV, WNV and BSQV, 1.6×10^5^ Vero B4 cells for DENV-2 and ROCV, and 1.2×10^5^ BHK-21 cells for SLEV. Forty plaque-forming units were incubated with respective serum dilutions of 1 : 40, 1:80, 1:160, 1:320, 1:640 and 1:1280 for 1 h, added onto the cell monolayer, and incubated for 1 h before adding an overlayer containing DMEM with 2% FCS and 1.25% carboxymethyl cellulose. Cells were incubated for 3 days for WNV and SLEV, 4 days for ZIKV, 5 days for SPOV, DENV-2 and BSQV, 6 days for ROCV and WSLV and 7 days for YFV. The overlayer medium was removed, and cells were fixated with 6% paraformaldehyde and stained with crystal violet. PRNT_90_ endpoint reciprocal titres were calculated using a logistic regression function in GraphPad Prism 6 (GraphPad Software, www.graphpad.com).

The initial ECLIA-based ZIKV reactivity rate among pets and peri-domestic animals was 4.6% (65/1416, 95% CI, 3.5–5.8) in Brazil and 3.6% (8/220, 95% CI, 1.6–7.0) in Côte d’Ivoire (Fig. 1, Table 1). However, based on PRNT_90_ fourfold titre differences, commonly considered decisive in flavivirus serology [42], ZIKV-specific antibodies could neither be confirmed in pets and peri-domestic animals from Brazil, nor from Côte d'Ivoire (Fig. 2a; Fig. 2b). Instead, among the Brazilian ECLIA-reactive sera, reciprocal PRNT_90_ endpoint titres were fourfold or higher for WNV in five (7.7%), YFV in three (4.6%), SPOV in one (1.5%) and WSLV in one serum (1.5%). A total of 31 ECLIA-positive sera (47.7% of all ZIKV ECLIA-reactive sera from Brazil) were negative for all flaviviruses tested by PRNT_90_, likely due to differential sensitivity of those tests. Among the eight ECLIA-reactive sera from pets and peri-domestic animals from Côte d'Ivoire, we found a monotypic reaction to YFV in one serum and to SPOV in another serum (Table S1, available in the online version of this article).

**Fig. 1.**
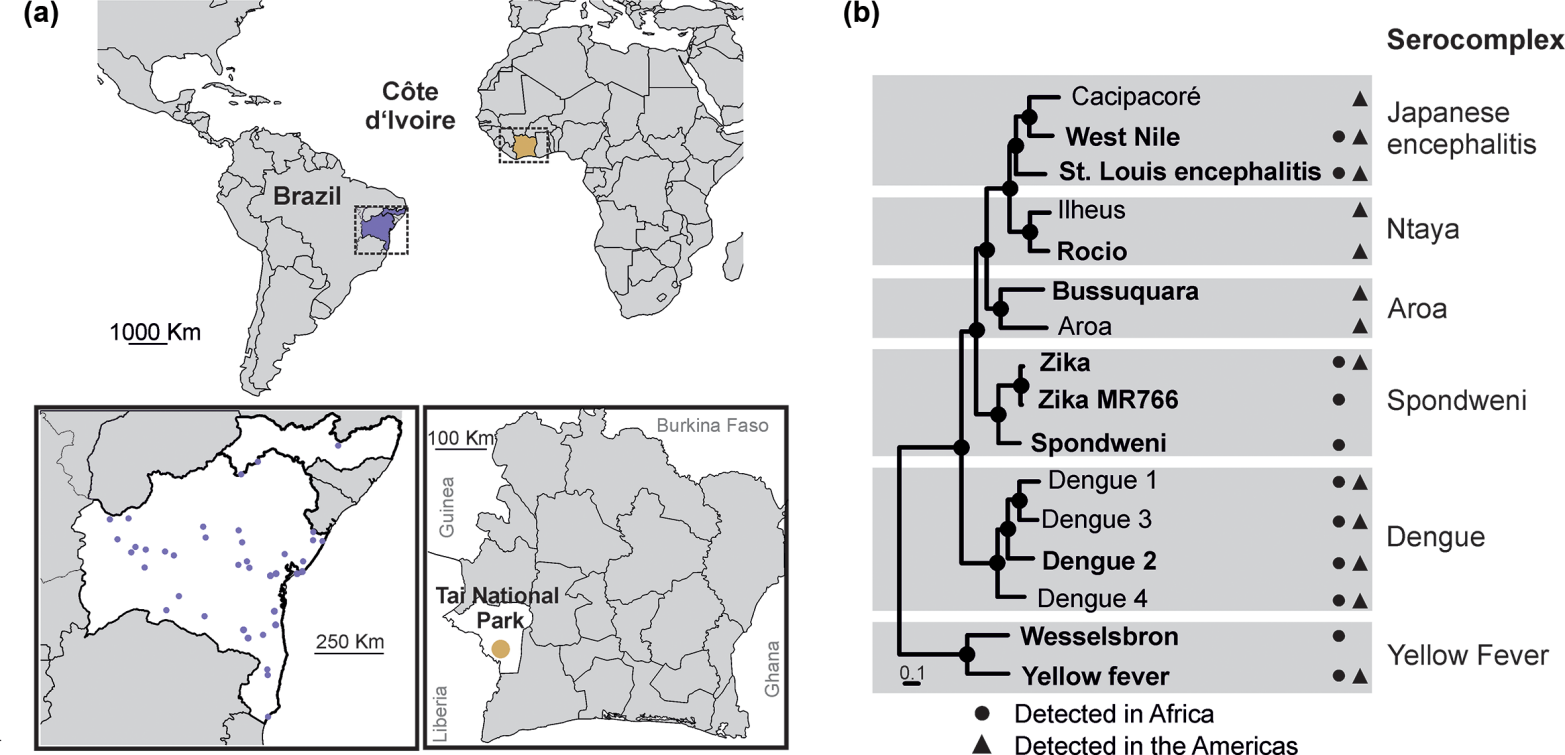
Sampling sites and endemic flaviviruses. Sampling sites in Brazil and in Côte d’Ivoire (a). Maximum-likelihood phylogeny of flaviviruses relevant for this study inferred using a dataset containing translated polyprotein genes and a Whelan and Goldman amino acid substitution model. Black circles at nodes represent support of grouping of ≥0.75 from 1000 bootstrap replicates. Viruses that were used in this study are highlighted in bold (b).

**Table 1.** Sampling table and Zika virus antibody test reactivity rate

Country sampled	Group	Animal species	Total	ECLIA % (95% CI)	PRNT_90_ % (95% CI)	Year
	Peri-domestic	Donkey	55	9.1 (3.0–19.9)	0	2013–2018
	Peri-domestic	Mule	95	4.2 (1.2–10.4)	0	2014–2018
	Peri-domestic	Horse	620	4.0 (2.6–5.9)	0	2015–2018
**Brazil**	Peri-domestic	Cow	278	6.5 (3.9–10.0)	0	2016–2018
	Peri-domestic	Sheep	64	4.7 (1.0–13.1)	0	2017, 2018
	Peri-domestic	Goat	231	2.6 (1.0–5.6)	0	2018
	Pet	Cat	5	20.0 (0.5–71.6)	0	2015, 2018
	Pet	Dog	68	4.4 (0.9–12.4)	0	2015, 2018
	Peri-domestic	Cow	16	12.5 (1.6–38.4)	6.3 (0.2–30.2)	2012–2014
	Peri-domestic	Goat	97	3.1 (0.6–8.8)	1.0 (0.1–5.6)	2012–2014
	Peri-domestic	Pig	18	0.0	0	2012–2014
	Peri-domestic	Sheep	89	2.2 (0.3–7.8)	0	2012–2014
**CIV**	NHP	King colobus (*Colobus polycomos*)	9	66.7 (29.9–92.5)	11.1 (0.3–48.3)	2006–2016
	NHP	Western red colobus (*Piliocolobus badius*)	13	23.1 (5.0–53.8)	0	2006–2016
	NHP	Sooty mangabey (*Cercocebus atys*)	17	0.0	0	2006–2016
	NHP	Chimpanzees (*Pan troglodytes verus*)	9	100.0 (66.4–100.0)	22.2(2.8–60.1)	2006–2016
	Pet	Dog	39	2.6 (0.1–13.5)	0	2006–2016
	**Total**		1723	5.3 (4.1–6.1)		

Detailed information is available in supplementary table S1.

CI, confidence intervals; ECLIA, electrochemiluminescence immunoassay; NHP, non-human primates; PRNT, plaque reduction neutralization test.

**Fig. 2.**
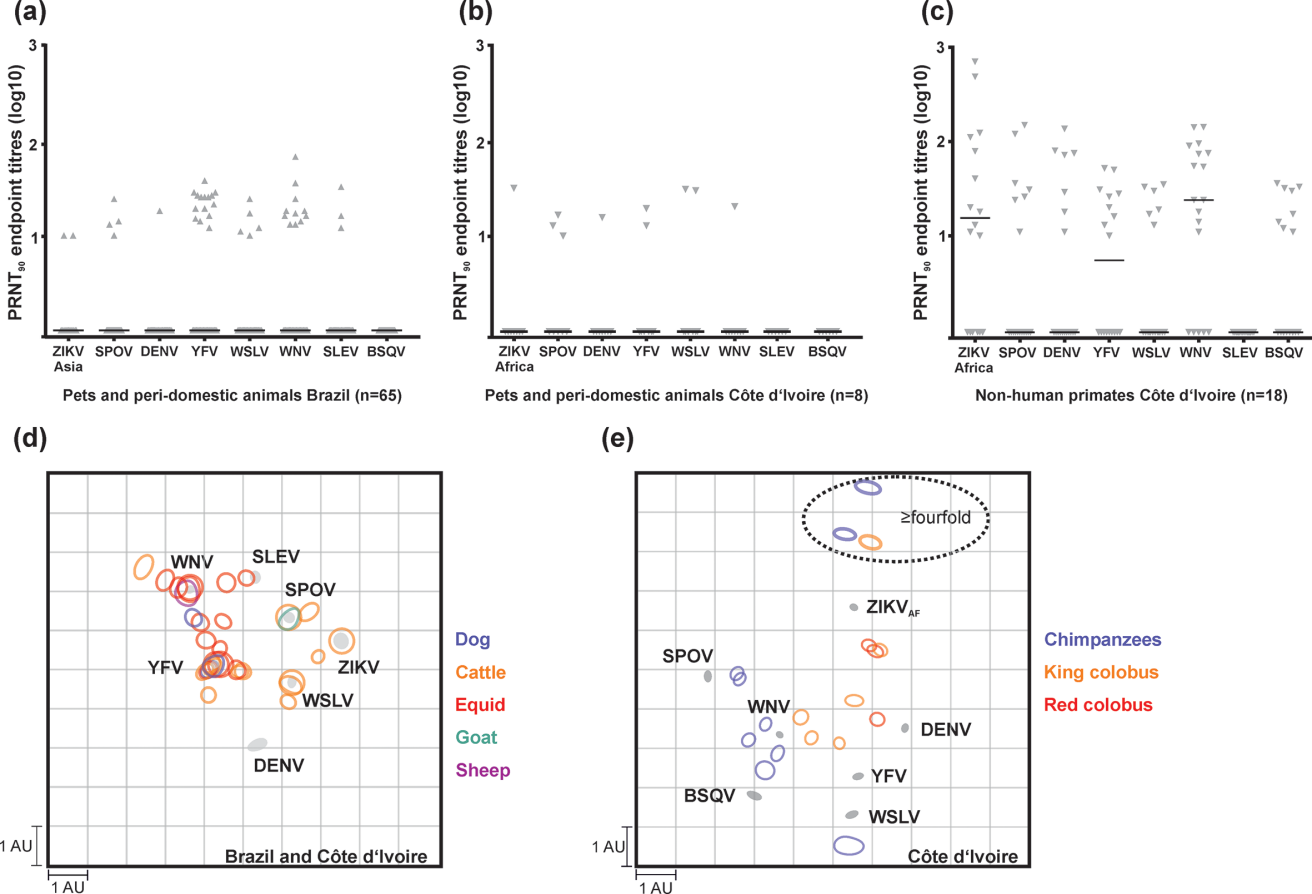
Flavivirus antibody reactivity patterns. Reciprocal PRNT_90_-specific endpoint titres for the African ZIKV lineage strain (ZIKV_AF_), the Asian ZIKV lineage strain (ZIKV_AS_), SPOV, DENV-2, YFV, WSLV, WNV, SLEV, ROCV and BSQV of ZIKV among ECLIA-positive sera in pets and peri-domestic animals in Brazil (a), in Côte d’Ivoire (b) and in NHP from Côte d’Ivoire (c). 2D antigenic cartography showing neutralizing activity against related flaviviruses among domestic animals in Brazil (d), Côte d’Ivoire (d) and in NHP in Côte d’Ivoire (e). Each unit of antigenic distance (length of one square grid side, measured in any direction) is equivalent to a fourfold dilution in the PRNT_90_. Each circle corresponds to one tested serum sample showing titres (sera with a negative PRNT_90_ or an endpoint titre <10 are not shown) and circle size suggests intra-sample differences. Grey circles indicate the antigens or antisera (tested viruses). In (d), titers obtained with the African ZIKV lineage strain were used for animals from Côte d’Ivoire and with the Asian ZIKV lineage strain for animals from Brazil. Horizontal lines plotted in (a), (b) and (c) show median

Antigenic cartography did not provide a robust separation of flavivirus serocomplexes in pets and peri-domestic animals from either Côte d`Ivoire or Brazil (Fig. 2d). The detection of sera with monotypic reaction or titres ≥fourfold for SPOV, and WSLV in Brazilian animals must be carefully interpreted. WSLV has not been reported in the Americas, and although SPOV has been isolated in *Culex quinquefasciatus* in Haiti [44] it is unlikely that either SPOV or WSLV would be widely dispersed among pets or peri-domestic animals from Brazil because there are no robust data on isolation or molecular detection of those viruses in vertebrates from Latin America. A more plausible explanation for those PRNT results are cross-reactive flavivirus antibodies, which complicate the interpretation of flavivirus serological assays in hyperendemic settings, even when considering only ≥fourfold PRNT_90_ titres as decisive serological support [42, 45]. This interpretation is supported by the overall low titres against all tested flaviviruses and by previous evidence showing the difficulties to confirm human ZIKV infection in DENV-endemic areas [42, 46].

In NHP, ZIKV ECLIA reactivity was observed in 23.1% (3/13, 95% CI, 11.1–39.3) of red colobus, 66.7% (6/9, 95% CI, 29.9–92.5) of king colobus and in none of the 17 sooty mangabeys, while 88.9% (8/9, 95% CI, 51.8–99.7) of the chimpanzees tested positive for ZIKV_AF_ using PRNT_90_. Fourfold or higher ZIKV PRNT_90_ titres compared to other flaviviruses were found in 11.1% (1/9, 95% CI, 0.3–48.3) of king colobus and in 22.2% (2/9, 95% CI, 2.8–60.1) of chimpanzees (Table 1, Fig. 2c, e) and in none of the previously ECLIA-reactive red colobus. Moreover, one chimpanzee (1/9, 95% CI, 0.3–48.3) presented titres ≥fourfold for WNV compared to all other flaviviruses. The difference in NHP ZIKV seroprevalence might be related to differential host susceptibility or different ecological niches occupied by those NHP since the mosquito species from which ZIKV was isolated in Côte d’Ivoire (i.e. *Aedes vitattus*, *Ae. furcifer* and *Ae. aegypti formosus*) are predominantly found in the tree canopy [28, 35, 47]. Notably, ZIKV was linked to NHP already in the initial viral isolation from a sentinel Rhesus macaque caged in the canopy [48]. Our data suggest that some NHP species like chimpanzees and king colobus, which are at least partly arboreal, might play a role as ZIKV reservoirs in Côte d’Ivoire. Our interpretation is supported by previous studies indicating ZIKV exposure of native African NHP by viral isolation from Patas (*Erythrocebus patas*) and Vervet (*Chlorocebus pygerythrus*) monkeys in Senegal [49] and by the detection of ZIKV-neutralizing antibodies in different NHP species such as in red-tailed monkey (*Cercopithecus ascanius*), grey-cheeked mangabey (*Lophocebus albigena*), mantled guereza (*Colobus guereza*) in Uganda [50], and tantalus (*Chlorocebus tantalus*) and Mona (*Cercopithecus mona*) monkey in Nigeria [51]. On the other hand, our data do not provide conclusive evidence on whether those NHP species may serve as amplification or reservoir hosts, because of our small sample size and because our methods were not targeted towards detecting active infections. Future longitudinal studies including younger NHP, as performed for CHIKV reservoir studies in Senegal [52], could clarify the role of those NHP species for ZIKV transmission in Africa.

Our study was limited by the lack of longitudinal sampling from the different animal species in both locations in Brazil and Côte d’Ivoire, since long-term titre comparison would be more effective for understanding antibody responses after multiple flavivirus infections. We also did not test other synanthropic animals such as marsupials, known to be relevant arbovirus hosts, e.g. for the alphavirus Ross River virus [53] and which may be exposed to ZIKV vectors [54]. However, while most of the previous studies on ZIKV animal reservoirs either used tests with lower specificity only (e.g. ELISA, complement fixation and haemagglutination) or assessed flaviviral cross-reactivity using fewer viruses in PRNT [27, 50, 51], we provided data from a large number of sera of pets and peri-domestic animals from two continents and performed extensive neutralization testing, considered the gold-standard technique.

In brief, our data confirm that co-circulating flaviviruses challenge unambiguous ZIKV antibody test results in hyper-endemic areas [41, 42]. Despite those technical limitations, our data imply that while some NHP species in Côte d’Ivoire may serve as ZIKV reservoir hosts, pets and peri-domestic animals are neither involved in ZIKV transmission cycles in Brazil, nor in Côte d’Ivoire.

## Supplementary Data

Supplementary material 1
