# Supplementary material for: Evidence against Zika virus infection of pets and peri-domestic animals in Latin America and Africa
Source: J Gen Virol. 2022 Jan 25;103(1):001709. doi: 10.1099/jgv.0.001709 (PMC8895617; doi:10.1099/jgv.0.001709)
Supplement: Supplementary material 1 [file jgv-103-1709-s001.pdf]

Supplementary Table 1. Comparative plaque-reduction neutralization test results for Zika virus ECLIA-positive serum samples from pets, peri-domestics and non-human primates

ID	Mosquito borne Flavivirus Serocomplex										Interpretation $PRNT_{90}$ *	Species	Year	Country
	Spondweni			Dengue	Yellow Fever		JE		Aroa	Ntaya				
	$ZIKV_{AS}$	$ZIKV_{AF}$	SPOV	DENV	YFV	WSLV	WNV	SLEV	BSQV	ROCV				
ZS-A-1	<10	N.A.	<10	<10	<10	<10	<10	<10	<10	<10	Negative	Horse	2013	Brazil
ZS-A-110	<10	N.A.	<10	<10	<10	<10	<10	<10	<10	<10	Negative	Horse	2014	Brazil
ZS-A-191	<10	N.A.	<10	<10	<10	<10	<10	<10	<10	<10	Negative	Horse	2013	Brazil
ZS-A-202	<10	N.A.	<10	<10	<10	<10	<10	<10	<10	<10	Negative	Horse	2013	Brazil
ZS-A-284	<10	N.A.	<10	<10	<10	<10	<10	<10	<10	<10	Negative	Goat	2013	Brazil
ZS-A-333	<10	N.A.	<10	<10	<10	<10	<10	<10	<10	<10	Negative	Goat	2016	Brazil
ZS-A-364	<10	N.A.	<10	<10	<10	<10	<10	<10	<10	<10	Negative	Goat	2016	Brazil
ZS-A-416	<10	N.A.	<10	<10	<10	<10	<10	<10	<10	<10	Negative	Goat	2016	Brazil
ZS-A-422	<10	N.A.	<10	<10	<10	<10	<10	<10	<10	<10	Negative	Goat	2016	Brazil
ZS-A-423	<10	N.A.	<10	<10	<10	<10	<10	<10	<10	<10	Negative	Goat	2016	Brazil
ZS-A-485	<10	N.A.	<10	<10	<10	<10	<10	<10	<10	<10	Negative	Horse	2014	Brazil
ZS-A-583	<10	N.A.	<10	<10	<10	<10	<10	<10	<10	<10	Negative	Horse	2013	Brazil
ZS-A-656	<10	N.A.	<10	<10	<10	<10	<10	<10	<10	<10	Negative	Donkey	2016	Brazil
ZS-A-660	<10	N.A.	<10	<10	<10	<10	<10	<10	<10	<10	Negative	Donkey	2017	Brazil
ZS-A-695	<10	N.A.	<10	<10	<10	<10	<10	<10	<10	<10	Negative	Cow	2014	Brazil
ZS-A-901	<10	N.A.	<10	<10	<10	<10	<10	<10	<10	<10	Negative	Cow	2016	Brazil
ZS-A-938	<10	N.A.	<10	<10	<10	<10	<10	<10	<10	<10	Negative	Cow	2016	Brazil
ZS-A-961	<10	N.A.	<10	<10	<10	<10	<10	<10	<10	<10	Negative	Cow	2016	Brazil
ZS-A-751	<10	N.A.	<10	<10	<10	<10	<10	<10	<10	<10	Negative	Horse	2013	Brazil
ZS-A-835	<10	N.A.	<10	<10	<10	<10	<10	<10	<10	<10	Negative	Horse	2017	Brazil
ZS-A-1224	<10	N.A.	<10	<10	<10	<10	<10	<10	<10	<10	Negative	Horse	2018	Brazil
ZS-A-1295	<10	N.A.	<10	<10	<10	<10	<10	<10	<10	<10	Negative	Horse	2018	Brazil
ZS-A-1308	<10	N.A.	<10	<10	<10	<10	<10	<10	<10	<10	Negative	Horse	2018	Brazil
ZS-A-1360	<10	N.A.	<10	<10	<10	<10	<10	<10	<10	<10	Negative	Horse	2018	Brazil
ZS-A-1361	<10	N.A.	<10	<10	<10	<10	<10	<10	<10	<10	Negative	Horse	2018	Brazil
ZS-A-1371	<10	N.A.	<10	<10	<10	<10	<10	<10	<10	<10	Negative	Horse	2018	Brazil
ZS-A-1413	<10	N.A.	<10	<10	<10	<10	<10	<10	<10	<10	Negative	Dog	2015	Brazil
ZS-A-1430	<10	N.A.	<10	<10	<10	<10	<10	<10	<10	<10	Negative	Dog	2018	Brazil

ZS-A-1433	<10	N.A.	<10	<10	<10	<10	<10	<10	<10	<10	Negative	Dog	2018	Brazil
ZS-A-1452	<10	N.A.	<10	<10	<10	<10	<10	<10	<10	<10	Negative	Cat	2018	Brazil
ZS-A-724	<10	N.A.	10	<10	<10	<10	<10	<10	<10	<10	SPOV	Cow	2016	Brazil
ZS-A-188	<10	N.A.	<10	<10	14	<10	<10	<10	<10	<10	YFV	Horse	2013	Brazil
ZS-A-255	<10	N.A.	<10	<10	16	<10	<10	<10	<10	<10	YFV	Horse	2013	Brazil
ZS-A-581	<10	N.A.	<10	<10	<10	<10	16	<10	<10	<10	WNV	Sheep	2017	Brazil
ZS-A-643	<10	N.A.	<10	<10	25	<10	<10	<10	<10	<10	YFV	Donkey	2016	Brazil
ZS-A-862	<10	N.A.	<10	<10	19	12	<10	<10	<10	<10	Undetermined	Cow	2016	Brazil
ZS-A-872	<10	N.A.	<10	18	37	<10	<10	<10	<10	<10	Undetermined	Cow	2016	Brazil
ZS-A-885	<10	N.A.	<10	<10	28	24	<10	<10	<10	<10	Undetermined	Cow	2016	Brazil
ZS-A-933	10	N.A.	24	<10	<10	<10	<10	<10	<10	<10	SPOV	Cow	2016	Brazil
ZS-A-952	<10	N.A.	<10	<10	26	<10	<10	<10	<10	<10	YFV	Cow	2016	Brazil
ZS-A-1005	<10	N.A.	<10	<10	19	10	<10	<10	<10	<10	Undetermined	Cow	2016	Brazil
ZS-A-728	10	N.A.	<10	<10	<10	<10	<10	<10	<10	<10	Negative	Cow	2016	Brazil
ZS-A-735	<10	N.A.	<10	<10	<10	11	<10	<10	<10	<10	WSLV	Cow	2016	Brazil
ZS-A-1123	<10	N.A.	<10	<10	<10	<10	14	16	<10	<10	Undetermined	Mule	2013	Brazil
ZS-A-1187	<10	N.A.	<10	<10	15	<10	18	<10	<10	<10	Undetermined	Horse	2018	Brazil
ZS-A-1226	<10	N.A.	<10	<10	25	<10	<10	12	<10	<10	Undetermined	Horse	2018	Brazil
ZS-A-1320	<10	N.A.	14	<10	<10	<10	17	32	<10	<10	Undetermined	Horse	2018	Brazil
ZS-A-1353	<10	N.A.	<10	<10	21	<10	13	<10	<10	<10	Undetermined	Horse	2018	Brazil
ZS-A-600	<10	N.A.	<10	<10	<10	<10	16	<10	<10	<10	WNV	Horse	2013	Brazil
ZS-A-1036	<10	N.A.	<10	<10	<10	<10	66	<10	<10	<10	WNV	Cow	2016	Brazil
ZS-A-1127	<10	N.A.	<10	<10	<10	<10	13	<10	<10	<10	WNV	Mule	2013	Brazil
ZS-A-1271	<10	N.A.	<10	<10	<10	<10	35	<10	<10	<10	WNV	Horse	2018	Brazil
ZS-A-1369	<10	N.A.	<10	<10	<10	<10	24	<10	<10	<10	WNV	Horse	2018	Brazil
ZS-A-684	<10	N.A.	<10	<10	<10	17	<10	<10	<10	<10	WSLV	Cow	2014	Brazil
ZS-A-511	<10	N.A.	<10	<10	25	<10	<10	<10	<10	<10	YFV	Sheep	2016	Brazil
ZS-A-911	<10	N.A.	<10	<10	26	<10	<10	<10	<10	<10	YFV	Cow	2016	Brazil
ZS-A-718	<10	N.A.	<10	<10	28	<10	<10	<10	<10	<10	YFV	Cow	2016	Brazil
ZS-A-58	<10	N.A.	<10	<10	<10	<10	<10	<10	<10	<10	Negative	Horse	2013	Brazil
ZS-A-161	<10	N.A.	<10	<10	<10	<10	<10	<10	<10	<10	Negative	Horse	2013	Brazil
ZS-A-254	<10	N.A.	<10	<10	<10	<10	<10	<10	<10	<10	Negative	Horse	2013	Brazil
ZS-A-516	<10	N.A.	<10	<10	<10	<10	<10	<10	<10	<10	Negative	Sheep	2016	Brazil

ZS-A-604	<10	N.A.	<10	<10	<10	<10	<10	<10	<10	<10	Negative	Horse	2013	Brazil
ZS-A-649	<10	N.A.	<10	<10	<10	<10	<10	<10	<10	<10	Negative	Donkey	2016	Brazil
ZS-A-997	<10	N.A.	<10	<10	<10	<10	<10	<10	<10	<10	Negative	Cow	2016	Brazil
ZS-A-1233	<10	N.A.	13	<10	12	<10	18	<10	<10	<10	Undetermined	Horse	2018	Brazil
SGAH369	N.A.	<10	<10	<10	21	<10	<10	<10	<10	N.A.	YFV	Sheep	2012	Côte d'Ivoire
SKEI478	N.A.	<10	<10	<10	<10	<10	<10	<10	<10	N.A.	Negative	Sheep	2012	Côte d'Ivoire
CoPAU293	N.A.	34	18	<10	<10	32	<10	<10	<10	N.A.	Undetermined	Cow	2012	Côte d'Ivoire
CoPAU317	N.A.	<10	11	17	<10	33	<10	<10	<10	N.A.	Undetermined	Cow	2012	Côte d'Ivoire
GGAH386	N.A.	<10	<10	<10	<10	<10	<10	<10	<10	N.A.	Negative	Goat	2012	Côte d'Ivoire
GZAI426	N.A.	<10	14	<10	<10	<10	<10	<10	<10	N.A.	SPOV	Goat	2012	Côte d'Ivoire
GPON024	N.A.	<10	<10	<10	<10	<10	<10	<10	<10	N.A.	Negative	Goat	2012	Côte d'Ivoire
DOG 17	N.A.	<10	<10	<10	14	<10	22	<10	<10	N.A.	Undetermined	Dog	2012	Côte d'Ivoire
C.b. 1	N.A.	11	<10	29	26	<10	<10	<10	<10	N.A.	Undetermined	Western red colobus (*Piliocolobus badius*)	2006	Côte d'Ivoire
C.b. 3	N.A.	79	24	136	<10	13	<10	<10	<10	N.A.	Undetermined	Western red colobus (*Piliocolobus badius*)	2007	Côte d'Ivoire
C.b. 8	N.A.	111	<10	75	<10	<10	24	<10	<10	N.A.	Undetermined	Western red colobus (*Piliocolobus badius*)	2007	Côte d'Ivoire
C. polyk 6	N.A.	10	<10	80	<10	17	95	<10	<10	N.A.	Undetermined	King colobus (*Colobus polycomos*)	2007	Côte d'Ivoire
C. polyk 9	N.A.	<10	<10	11	52	<10	54	<10	14	N.A.	Undetermined	King colobus (*Colobus polycomos*)	2007	Côte d'Ivoire
C. polyk 10	N.A.	20	<10	18	31	33	24	<10	11	N.A.	Undetermined	King colobus (*Colobus polycomos*)	2007	Côte d'Ivoire
C. polyk 2	N.A.	123	<10	72	16	<10	11	<10	<10	N.A.	Undetermined	King colobus (*Colobus polycomos*)	2007	Côte d'Ivoire
C. polyk 3	N.A.	487	<10	<10	10	<10	18	<10	<10	N.A.	$ZIKV_{AF}$	King colobus (*Colobus polycomos*)	2007	Côte d'Ivoire
C. polyk 4	N.A.	<10	<10	<10	50	<10	90	<10	<10	N.A.	Undetermined	King colobus (*Colobus polycomos*)	2007	Côte d'Ivoire
Chimp 20	N.A.	18	120	<10	<10	<10	74	<10	12	N.A.	Undetermined	Chimpanzees (*Pan troglodytes verus*)	2006-2016	Côte d'Ivoire
Chimp 76	N.A.	40	149	<10	<10	<10	141	<10	33	N.A.	Undetermined	Chimpanzees (*Pan troglodytes verus*)	2006-2016	Côte d'Ivoire
Chimp 365	N.A.	1030	11	<10	<10	<10	<10	<10	<10	N.A.	$ZIKV_{AF}$	Chimpanzees (*Pan troglodytes verus*)	2006-2016	Côte d'Ivoire
Chimp 6	N.A.	702	31	<10	28	<10	<10	<10	<10	N.A.	$ZIKV_{AF}$	Chimpanzees (*Pan troglodytes verus*)	2006-2016	Côte d'Ivoire
Chimp 560	N.A.	13	26	<10	<10	19	75	<10	36	N.A.	Undetermined	Chimpanzees (*Pan troglodytes verus*)	2006-2016	Côte d'Ivoire

Chimp 558	N.A.	<10	<10	<10	<10	<10	14	<10	17	N.A.	Undetermined	Chimpanzees (*Pan troglodytes verus*)	2006-2016	Côte d'Ivoire
Chimp 429	N.A.	<10	<10	<10	20	<10	55	<10	32	N.A.	Undetermined	Chimpanzees (*Pan troglodytes verus*)	2006-2016	Côte d'Ivoire
Chimp 557	N.A.	<10	<10	<10	<10	30	<10	<10	<10	N.A.	WSLV	Chimpanzees (*Pan troglodytes verus*)	2006-2016	Côte d'Ivoire
Chimp 1900	N.A.	<10	36	<10	13	35	142	<10	30	N.A.	WNV	Chimpanzees (*Pan troglodytes verus*)	2006-2016	Côte d'Ivoire

ZIKV$_{AS}$, Zika virus Asian Strain; ZIKV$_{AF}$, ZIKV virus African strain; SPOV, Spondweni virus; DENV, Dengue virus; YFV, Yellow Fever virus; WSLV, Wesselsbron virus; WNV, West Nile virus; SLEV, Saint Louis Encephalitis virus; BSQV, Bussuquara virus; ROCV, Rocio virus; PRNT, Plaque neutralization assay test; N.A. not available; * determined as fourfold or higher PRNT$_{90}$ endpoint titers
